# Low-resistance monovalent-selective cation exchange membranes prepared using molecular layer deposition for energy-efficient ion separations†

**DOI:** 10.1039/d0ra08725d

**Published:** 2021-01-11

**Authors:** Eyal Merary Wormser, Oded Nir, Eran Edri

**Affiliations:** Blaustein Institutes for Desert Research, Zuckerberg Institute for Water Research, Ben-Gurion University of the Negev Sede Boqer Campus 8499000 Israel edrier@bgu.ac.il; Department of Chemical Engineering, Ben-Gurion University of the Negev Beer-Sheva 8410501 Israel

## Abstract

The desalination of brackish water provides water to tens of millions of people around the world, but current technologies deplete much needed nutrients from the water, which is determinantal to both public health and agriculture. A selective method for brackish water desalination, which retains the needed nutrients, is electrodialysis (ED) using monovalent-selective cation exchange membranes (MVS-CEMs). However, due to the trade-off between membrane selectivity and resistance, most MVS-CEMs demonstrate either high transport resistance or low selectivity, which increase energy consumption and hinder the use of such membranes for brackish water desalination by ED. Here, we introduce a new method for fabrication of MVS-CEMs, using molecular layer deposition (MLD) to coat CEMs with ultrathin, hybrid organic–inorganic, positively charged layers of alucone. Using MLD enabled us to precisely control and minimize the selective layer thickness, while the flexibility and nanoporosity of the alucone prevent cracking and delamination. Under conditions simulating brackish water desalination, the modified CEMs provides monovalent selectivity with negligible added resistance—thereby alleviating the selectivity–resistance trade-off. Addressing the water-energy nexus, MLD-coating enables selective brackish water desalination with minimal increase in energy consumption and opens a new path for tailoring membranes' surface properties.

## Abstract 2

The desalination of brackish water provides water to tens of millions of people around the world, but current technologies deplete much needed nutrients from the water, which is determinantal to both public health and agriculture.

## Graphical abstract

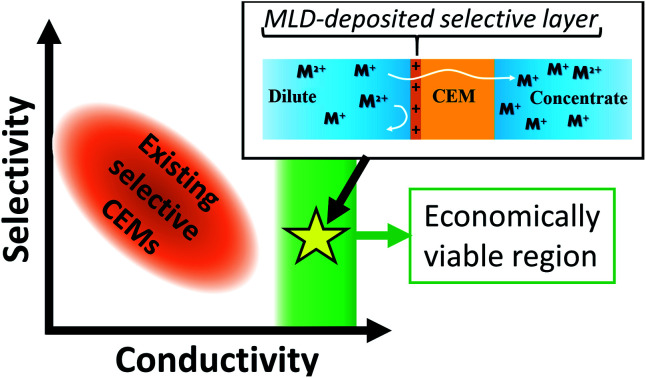

## 1. Introduction

Water scarcity affects most of the world population^1,2^ and is expected to increase with the rise in population size and quality of life.^2^ Desalination, the process of removing salts from water, is becoming a vital part of the solution to water scarcity,^3^ with a current worldwide desalination capacity of approx. 100 million m^3^ per day, mostly for domestic use,^4^ which is expected to grow by 40% by 2030. In 2019, 29% of the global desalination capacity was of brackish and river water (500–20 000 ppm TDS),^4^ which provided water to tens of millions of people worldwide. Most (98%) of the brackish water nowadays is desalinated by reverse osmosis,^5^ which completely deprives the permeate of Mg^2+^ and Ca^2+^. Both are critical nutrients needed to sustain human health^6^ and agriculture.^7^ Replenishing these divalent ions to the desalinated water increases the cost and complexity of the overall process and decreases its sustainability and economic attractiveness. A selective desalination technique—which can retain these ions in adequate concentrations while removing undesirable monovalent ions (mainly Na^+^ and Cl^−^ in the context of brackish water desalination)—is a timely goal for research.^8^ In other contexts, selective concentration of useful monovalent ions such as Li^+^ can be the motivation for selective desalination.^8,9^ A possible, cost-effective solution is to combine monovalent-selective cation-exchange membranes (MVS-CEMs) in electrodialysis to facilitate monovalent selective electrodialysis, which is a promising alternative for brackish water reverse osmosis because of its higher energy efficiency^10^ and high recovery ratios.^4^ Using ED with MVS-CEMs could retain beneficial divalent cations in the product water while excluding monovalent ions (see ESI Section 1†).^11^ Nonetheless, monovalent selective ED is not widely used for brackish water desalination, partially because the MVS-CEMs have higher resistance than standard CEMs. While in this work we approach the water-energy-health nexus by focusing on the case study of selective brackish water desalination, selective ED is useful for both low-salinity operations (due to its high energy efficiency in low salinity)^12^ and high salinity (as it can achieve high recovery ratios and concentrate minerals to a high degree), leading to the use of MVS-CEMs in a variety of other processes such as treating and recovering valuable minerals from desalination brine^13,14^ and salt-lakes.^15,16^ MVS-CEMs are also used in novel processes such as selective capacitive deionization (CDI)^17^ and reverse electrodialysis (RED).^18–22^

Monovalent selectivity in CEM is almost exclusively instilled by adsorbing thin organic layers, most commonly a polyelectrolyte with an opposite charge to that of the CEM. However, to achieve a uniform coating of the CEM by the selective layer—needed to prevent non-selective ion transport pathways—multiple coating layers are applied, which results in tens of nanometers thick layers and increases transport resistance. New membranes that better balance the selectivity–resistance trade-off are, therefore, needed. A key element in reducing the resistance of MVS-CEMs is to minimize the thickness of the selectivity-inducing layer while retaining a uniform coverage.

Here, we introduce a new method for fabricating MVS-CEM for brackish water desalination, based on the deposition of a highly uniform, positively charged ultra-thin layer using molecular layer deposition^23^ (MLD) of Eg-alucone.^24^ In the past decade, atomic layer deposition (ALD) has become commercially available for coating polymers in a roll-to-roll processes,^25^ and it has been used to deposit thin layers on polymers for various purposes, including gas diffusion barriers^26^ and the encapsulation of flexible electronics.^27^ ALD was performed on various types of separation membranes,^28^ including for tailoring pore size, instilling ion selectivity, or altering the surface properties of reverse-osmosis (RO)^29,30^ and nanofiltration^31^ (NF) membranes. To the best of our knowledge, the use of neither ALD nor MLD has been reported for the coating of ion exchange membranes. Lab-scale ED experiments simulating brackish water desalination showed that the MLD treatment successfully instilled monovalent selectivity in a non-selective CEM. The selectivity was close to that of a commercially available selective membrane, but its area resistance – 2.42 Ω cm^2^ – was about half of the resistance of the commercially available MVS-CEM we tested. Notably, the increase in resistance attributed to the coating was ∼0.2 Ω cm^2^, which is the lowest added resistance reported to date for a monovalent-selective layer, and is small enough to allow usage of membranes coated by such a layer in place of standard CEMs without a major impact on energy consumption.

## 2. Materials and methods

### 2.1. Membrane coating

ALD and MLD were performed in a Gemstar XT™ benchtop ALD reactor. Al_2_O_3_ ALD was performed at 40 °C, by alternating exposures to a 21 ms pulse of trimethylaluminum (TMA; electronic grade STREM Co.), followed by a 60 s purge, a 21 ms pulse of H_2_O (deionized water with <5 ppm TOC and 18.2 Ω cm resistance), and another 60 s purge before the next cycle. Throughout the deposition process, Ar (99.999% pure; Maxima Ltd.) flowed through the reactor at 10 SCCM, serving as a carrier/purge gas. Base pressure in the reactor was ∼170 mTorr. MLD was performed at 65 °C using a similar protocol, with a 1 s pulse of ethylene glycol (>99% pure, Bio Lab) preheated to 65 °C to increase its vapor pressure. To prevent condensation, the manifold lines were heated to 130 °C (Eg/H_2_O) or 115 °C (TMA). *In situ* quartz crystal microbalance (QCM) was used to monitor the deposition process on 6 MHz Au-coated quartz crystals using Inficon SQM-160 monitor.

Ion exchange membranes (PC-SK, non-monovalent selective cation exchange membrane, and PC-MVK monovalent selective cation exchange membrane) were purchased from PCA-GmbH. Membrane specifications, as provided by the manufacturer at the time of purchase, are presented in Table 1. The membranes were delivered stored in 25% wt. NaCl. Before deposition, the PC-SK membranes were washed with deionized water, dried under a nitrogen stream (99.999% pure), and left to dry completely and equilibrate for 10 minutes at the deposition conditions inside the ALD reactor (170 mTorr; 65 °C). Si (100 orientation, B-doped) and microscope glass slide substrates, which were used for ellipsometry and surface-potential measurements, respectively, were cleaned for 1 h with a piranha solution (1 : 3 mixture of concentrated sulfuric acid and 33% wt. H_2_O_2_. CAUTION: exothermic reaction, perform in a fume hood and take safety measures to handle corrosive fumes and liquid) and dried with a nitrogen stream prior to coating.

**Table 1:** Manufacturer data for the commercial membranes used in this work

Membrane	Functional group	Transference number KCl (0.1/0.5 N)	Resistance [Ω cm^2^]	Water content [wt%]	Ion exch. capacity [meq. g^−1^]	Thickness [μm]	Reinforcement
PC-SK	Sulfonic acid	>0.95	∼2.5	∼9	3	160–200	Polyester
PC-MVK	Sulfonic acid	>0.97	n.a	n.a	n.a	100	PVC

### 2.2. Characterization

Scanning electron microscopy (SEM) was performed with a VERIOS XHR 460L, and samples were pre-coated with ∼5 nm Ir. Energy-dispersive X-ray spectroscopy (EDS) measurements were performed in the SEM by using an Oxford instrument X-MAX™ 80 detector, at an accelerating voltage of 5 keV and a probe current of 0.2 nA. Transmission electron microscopy (TEM) was performed using a Tecnai T12 TEM, and samples were pre-embedded in epoxy (epoxy embedding medium kit, Sigma-Aldrich) and sliced to ∼100 nm thick slices in a microtome.

The *ζ* potential was measured using the streaming potential method. Measurements were performed with an Anton Paar surPASS© 2 Electro Kinetic Analyzer, employing solutions of 0.01 M KCl. The pH of the solution was changed by adding NaOH or HCl. For each streaming potential measurement, the pressure was ramped from 0 to 400 mbar and held for 180 s. Each measurement was repeated twice in each flow direction. Notably, streaming potential measurements on a CEM led to erroneous results due to ion-exchange effects. The measurements presented in this work were performed on glass substrates placed in a clamping cell with a 100 μm flow gap. As a reference, a polypropylene sheet was used on the other side of the flow channel. The reference sheet streaming potential was measured separately and subtracted from the final results.

X-ray photoelectron (XPS) measurements were performed using an ESCALAB 250 XPS/AES on a ∼20 nm layer of alucone. The X-ray photons excited only the alucone layer and not the substrate, as verified by the lack of a S/Si signal (on PC-SK or glass substrates, respectively). All photoelectric peaks binding energy were manually scaled by setting the binding energy of C 1s C–H and C–C peak at 284.7 eV.

FT-IR spectra were collected with a Thermo Scientific Nicolet iS50R spectrometer. A Ge-ATR with a 60° cut was used in a Pike Technologies Veemax III variable angle accessory. Membranes were soaked in ultrapure double deionized water (18.2 Ω cm; <5 ppm TOC) during measurements and uniformly pressed against the ATR crystal with a constant force. The spectrometer and ATR accessory were continuously purged with 99.999% N_2_ during measurements. A DTGS detector was used to collect and average 128 scans at a resolution of 4 cm^−1^.

Differential Scanning Calorimetry (DSC) was performed in a Mettler Toledo Star DSC operated under a N_2_ flow of 80 mL min^−1^ and equipped with 70 μL alumina crucibles by heating the samples from 30 °C to 200 °C, cooling back to 30 °C, and heating again to 200 °C, all at a rate of 10 °C min^−1^.

### 2.3. Electrodialysis experiments and resistance measurements

Electrodialysis experiments were performed with a mixed solution of 1000 ppm Na^+^ (as Na_2_SO_4_, ≥99% purity, Merck) and 100 ppm Mg^2+^ (as MgSO_4_·7H_2_O, ≥99.5% purity, Merck) dissolved in deionized water (18.2 MΩ cm)—concentrations that are similar to those found in the brackish water of the Israel Negev region.^32^ ED was conducted at a constant current density of 2.5 mA cm^−2^, applied using a Lion LE 305D DC laboratory power supplier. A single cell-pair was used in the following configuration (illustrated in Fig. S1†): the tested membrane, with the treated (selective) side facing the anode, was placed between two PC-SA standard AEMs in a PCCell-GmbH micro-ED electrodialyser with a Pt/Ir-coated titanium anode and a V4A steel cathode. The dilute and concentrate were circulated at 8.5 mL min^−1^ from a 50 mL batch. One 200 mL batch of a 0.25 M Na_2_SO_4_ solution was used for both the cathode and anode, flowed at 50 mL min^−1^, and constantly mixed back into the batch to negate pH changes due to the electrode reactions. Each experiment lasted 60 min and reached ∼70% desalination (unless specified otherwise). Membranes were soaked in the feed solution to equilibrate for 24 h prior to the experiments. During the experiments, 50 μL aliquots were taken from the dilute and concentrate compartments at specific time intervals, diluted 20 times with deionized water, and their ion composition measured using a SPECTRO ARCOS ICP-OES in a SOP configuration. 
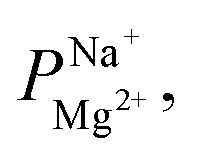
 the permselectivity of Na^+^ over Mg^2+^, was calculated according to the equation:1
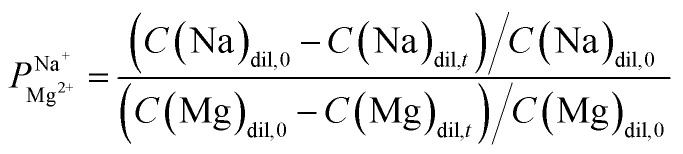
where *C*(X)_dil,0_ is the concentration of ion X in the dilute at the beginning of the experiment and *C*(X)_dil,0_ is its concentration at the specific sampling time. All selectivity values reported in this paper are an average of at least four fresh samples, and uncertainty values are the standard deviations of each set, with the exception of the cycling experiment, which was performed once and whose errors are based on the ICP measurement errors.

In the experiment performed at pH 9.5, the pH of the feed water was increased prior to ED by adding 0.1 M NaOH. Then, it was monitored and kept constant in the dilute compartment by manually adding 50 μL 0.1 M NaOH whenever the pH dropped by at least 0.25 pH units below the desired pH; the volume added to the solution and the Na^+^ concentration added to the feed were negligible (<1%).

The lack of standard conditions for testing permselectivity of counter ions limit the lab-to-lab comparison of selectivity values. Large variations, up to several orders of magnitude, are reported for membranes prepared by similar means.^33,34^ Moreover, variations by a factor of two can be found even for the same (commercially-available) membrane in different setups.^7,35^ These variations can be related to different process conditions and experimental conditions in different reports, highlighting the urgent need for standardization in the field of selective ion exchange membranes development. Until then, however, we find the best practice for testing new MVS-CEMs, is to compare a newly developed membrane with commercially available membranes (both selective and non-selective) using the same experimental setup and in conditions that simulate the desired application.

While ohmic resistance could, in principle, be calculated using the voltage measured during the ED experiments, the resolution of the measured voltage, in combination with the high resistance of the system, did not enable us to produce high-resolution measurements directly from these experiments, such that a separate set of measurements in a dedicated setup was required. Membrane resistance was measured at 25 °C using a standard conductivity meter (El-Hamma Instruments TH-2300 conductivity/temperature meter; measuring frequency 800 Hz) in 0.5 M NaCl solution, using a custom-made apparatus, based on the method and equipment described by Shapiro *et al.*^36^ Briefly, the apparatus included platinum black-coated Pt electrodes at both sides of a flow cell with fixed, known dimensions, such that the membrane was “sandwiched” in the middle of the cell. The cell conductivity was first measured without the membrane (with the solution flowing through the cell), which was then subtracted from the measurements of the membranes. The PC-SK membrane was dried under vacuum inside the ALD reactor at 65 °C and 40 °C, so as to negate the impact of these conditions on resistance and focus on the resistance added by the coating itself. Separate measurements were performed without drying so as to isolate the impact of drying. PC-MVK membranes were not dried prior to measurements. All membranes were equilibrated for at least 1 h in 0.5 M NaCl prior to measurement. Each measurement was performed at least three times using different membrane samples, and the errors presented in the article reflect the standard deviation between repetitions.

## 3. Results and discussion

### 3.1. Al_2_O_3_ ALD on CEMs

**Fig. 1:**
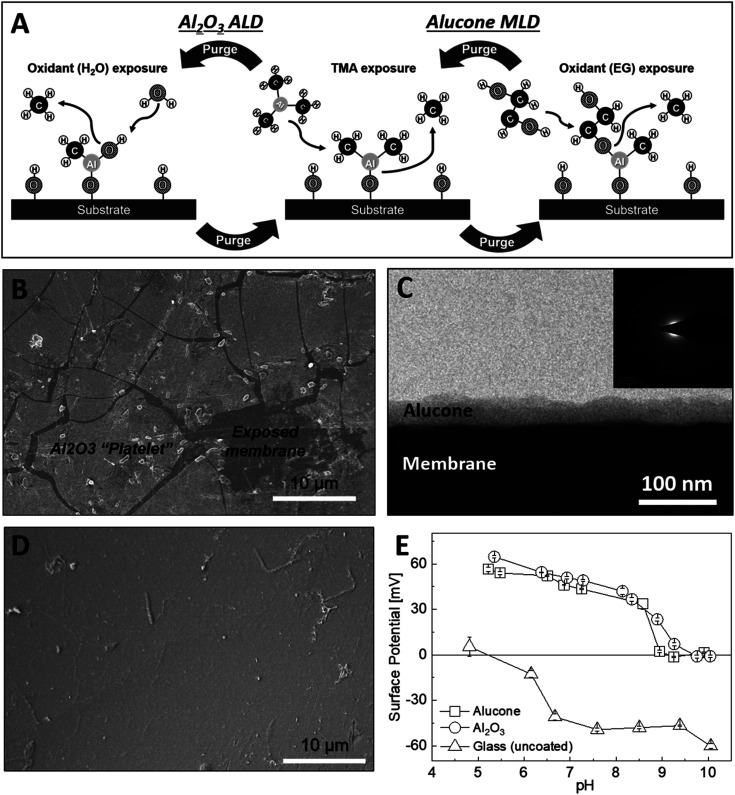
Structure and properties of ALD- and MLD-deposited compounds. (A) Schematic illustration of ideal reactions in Al_2_O_3_ ALD (left), performed using subsequent exposures to TMA and H_2_O, and alucone MLD (right), with TMA and EG. The subsequent exposures with the purge times in between them comprise one deposition cycle. (B and D) Plan view scanning electron micrographs of membranes after wetting in deionized water for 24 h. Evidently, the Al_2_O_3_ coating by 25 ALD cycles of a membrane (B) contains cracks and exposed CEM surface where the Al_2_O_3_ peeled, whereas the Eg-alucone coating of a membrane by 25 MLD cycles (D) is intact. (C) A cross-section TEM of a PC-SK CEM coated by 25 alucone cycles. The deposition of a highly conformal layer with a sharp interface with the substrate can be seen. The layer is approx. 30 nm thick and the average growth rate was approx. 1.2 nm per cycle. The inset demonstrates the absence of a crystalline diffraction pattern in a close-up image of the coating, indicating that the Eg-alucone layer is amorphous. (E) The surface potential of layers deposited by 100 ALD or MLD cycles on a glass substrate. Positive surface potentials up to the isoelectric point (IEP) at approx. 9 for alucone (triangles), 9.5 for Al_2_O_3_ (circles), and ∼5 for the uncoated glass substrate (squares) are seen. Error bars indicate the standard deviation of four measurements at each pH (two in each flow direction).

A major advantage of ALD for modifying the surface electrostatic charge density of membranes is the ability to deposit thin and conformal layers of metal oxides at low temperatures. For example, the deposition of Al_2_O_3_*via* ALD (Fig. 1A) is well established as low as 25 °C.^37,38^ Moreover, the high isoelectric point (IEP) of Al_2_O_3_ predicts a positive surface potential in neutral pH solutions, which makes Al_2_O_3_ a good candidate for instilling a charge-based exclusion of cations in CEMs. Indeed, using Al_2_O_3_ that was ALD-deposited at 65 °C, we were able to uniformly coat the PC-SK CEMs. However, when the modified CEM was hydrated for ED, the Al_2_O_3_ layer cracked and delaminated (Fig. 1B). We note that the Al_2_O_3_ layers did not show such a behavior on glass or Si substrates; thus, we attribute the instability of the Al_2_O_3_ ALD coating on the PC-SK membranes to the interaction with the supporting CEM. Specifically, we attribute it to the elastic mismatch between the rigid Al_2_O_3_ layer and the flexible polymer substrate, which focuses the mechanical stresses induced by the substrate swelling and contraction during wetting and drying, at the organic–inorganic interface. We resolved to remedy that issue by substituting the rigid Al–O–Al bonds with flexible organic linkers between adjacent Al atoms. Lee *et al.* previously found that alucone layers—hybrid organic–inorganic layers incorporating ethylene glycol instead of oxygen—are ∼12 times softer than alumina layers and have an ∼8 times smaller elastic modulus.^39^ Indeed, as demonstrated in the SEM in Fig. 1D, the alucone layers did not suffer from the instability observed in the Al_2_O_3_ layers (Fig. 1B) upon wetting and drying. Thus, the lower rigidity due to the inclusion of organic linkers in the MLD process appears to increase the stability of MLD layers deposited on PC-SK CEMs.

### 3.2. MLD of alucone on PC-SK CEM

Unlike the Al_2_O_3_ ALD, an alucone MLD (Fig. 1A) was previously reported only at temperatures above 80 °C.^40^ However, a DSC scan (Fig. S2†) of the polymeric base CEM (PC-SK CEM) revealed a glass transition at around ∼85 °C and a decomposition or melting of the polymer above ∼190 °C. Using a combination of *in situ* QCM (Fig. S3A†) and ellipsometry (Fig. S3B†), we developed an alucone MLD procedure at 65 °C. We found that, on hard and impermeable substrates such as Au-coated quartz or Si wafer, a constant alucone growth rate of 2.2 ± 0.1 Å per cycle is observed with a stepwise mass increase. As the expected growth rate for a fully extended ethylene glycol molecule in a single MLD cycle is ∼8.4 Å, we attribute the smaller observed growth rate to the bending and rotation of the ethylene glycol along C–C and C–O bonds. However, the linear and stepwise growth are hallmarks of ALD and MLD growth modes.

The deposition of alucone on a PC-SK membrane at 65 °C resulted in a conformal coating of the membrane (Fig. 1C), but the growth rate was 12–15 Å per cycle. This growth rate is significantly higher than the growth rate we obtained here for alucone on hard and impermeable substrates; and also higher than the expected growth rate for a fully extended ethylene glycol molecule in a single MLD cycle. Therefore, while QCM and ellipsometry data indicated that alucone deposition at 65 °C is within the so-called ‘ALD window’, the soft and permeable nature of the polymeric membrane appears to exert deviation from an ideal MLD growth mode. This is also evident in an enhanced growth rate (3.8 ± 0.3 Å/cycle) over a spectating Si wafer put together in the coating chamber with the membrane during MLD (Fig. S3B†). This possibly indicates an infiltration of precursors into the CEM, as was also reported for Al_2_O_3_ ALD on polymers.^41,42^ Nonetheless, EDS measurements of the alucone-coated CEMs (Fig. S3C;† averaged over several square millimeters) indicate that, in the range that we tested, the amount of Al increased linearly with the number of cycles in both Al_2_O_3_ ALD and alucone MLD.

**Fig. 2:**
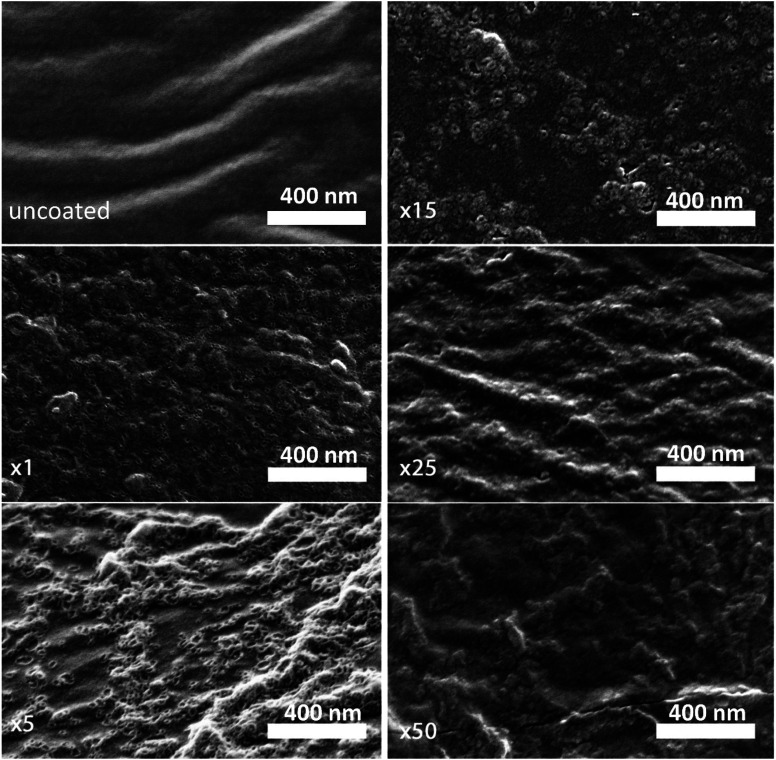
Scanning electron micrographs of the uncoated membrane (dried in the ALD reactor at the process conditions) and of the membrane after 1, 5, 15, 25, and 50 alucone MLD cycles. Dimples (20–30 nm) on the surface, which are not present in the uncoated membrane, can be seen to form immediately with the first coating cycle, and they are gradually covered by alucone, from the sides up, to form ‘alucone domes’ that close after around 25 deposition cycles. Images taken at a 65 000× magnification with a 30° sample tilt with respect to the in-lens detector.

A high-resolution SEM was used to examine the morphology of the deposited layer. As can be seen in Fig. 2, the initial alucone layer was not uniformly deposited on the PC-SK surface; rather, large sections were coated with continuous layers, while round, 20–30 nm sunken regions (‘dimples’) appeared to be uncoated. The absence of Al in these dimples and its presence in the surrounding regions were confirmed by a SEM-EDS elemental mapping (data not shown). Additionally, while dimples in the surface were discernable after a single MLD cycle, they were not observed in the pristine PC-SK membrane, indicating that they resulted from the MLD process, rather than from the drying and loading in a high vacuum. Adding more alucone layers resulted in the formation of ‘alucone domes’ over the dimples, namely, alucone coating over the dimples, and these domes appeared to close after ∼25 cycles of alucone deposition (Fig. 2). Additional coating layers seemed to uniformly thicken the alucone dome layer, while the dimpled areas mostly disappeared and the membrane surface appeared to be smooth. At a higher coating thickness, the coating was more prone to cracking (but not peeling), especially when the membrane was folded or handled roughly.

### 3.3. Properties of the alucone layer deposited on CEMs

When exposed to liquid water or ambient humidity, the alucone layer is known to undergo a chemical and morphological transformation and become a porous layer (∼40% porosity; with cylindrical pores, 5–8 nm in diameter) having a complex chemical composition.^43^ To better understand the composition and structure of the alucone layer deposited on the CEM, we examined the chemical composition of the surface using XPS (Fig. S5†) and ATR-FT-IR (Fig. S4†). XPS results of as-prepared alucone-coated CEMs indicate that the O/Al atomic ratio was 2.1. Following a 2 h exposure to water, the O/Al ratio increased to 2.4 and slight shifts were observed in the Al 2p and O 1s binding energy peaks. This deviation from the expected stoichiometry, along with other subtle changes (discussed in the ESI†), indicate the initial presence of unreacted methyl groups in the alucone layer, which react with water over time. The ATR-FT-IR analysis supported the existence of Al–O bonds and showed evidence of the existence of vinyl ether groups, which are unexpected in the ideal alucone structure but were previously reported to form during the post-deposition transformation of alucone under ambient conditions.^24,43^ A more detailed analysis of this transformation and the ATR-FT-IR spectra can be found in the ESI.† Since neither XPS nor ATR-FT-IR revealed direct evidence of a reaction of the MLD precursors with the CEM, we conclude that the chemical transformations of the alucone on the CEM is similar to the transformation that occur on other substrates. The chemical transformations amount to morphological changes in the alucone layer and facilitate the formation of pores, but we were unable to directly verify or refute the existence of such pores in the alucone deposited on a CEM. Such pore formation (and properties) should be further examined in future studies.

To serve as a selective layer based on coulombic repulsion, the surface potential of the coating should be positive, but, to date, this has not been established for alucone. To determine the IEP of the deposited layer, we conducted streaming potential measurements on a glass substrate coated with 100 cycles of alumina or alucone. The IEP of both alucone and alumina layers was 9–9.5 (Fig. 1E), which is significantly higher than the approximately neutral pH in typical brackish water desalinated for drinking or agriculture. A repeated measurement of the alucone layer's surface potential at pH 5 at the end of the experiment lead to a similar positive potential to that of the first measurement (not shown), indicating that the change in surface potential did not stem from instability at high pH. Therefore, we expect the alucone layer on the CEM to have a positive surface charge under standard ED operating conditions.

### 3.4. Performance factors for electrodialysis

Obtaining a target concentration of bivalent cations in the desalinated water, coupled to a target removal rate of monovalent cations, requires a minimal level of monovalent cation selectivity. To evaluate our membranes in this context, we considered brackish water desalination by ED (using the saline aquifer in the Negev, Israel as a benchmark), targeted at removing 90% of the monovalent ions, while retaining at least 20 mg L^−1^ of Mg^2+^ (recommended for drinking water). A simple mass-balance (detailed calculations are in the ESI†) revealed that the minimal required selectivity for attaining such water quality is 
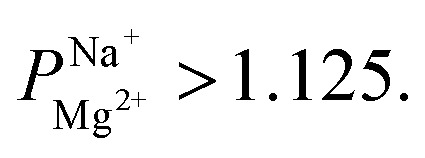
 While higher selectivity values might be beneficial, they are not strictly necessary and can be expected to come at a cost of added resistance.

We conducted a series of ED experiments designed to simulate selective brackish water desalination. The uncoated PC-SK membranes were not selective to ion transport 
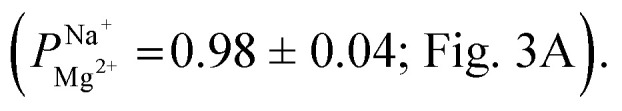
 Depositing 5, 25, or 50 cycles of alucone on the PC-SK membrane by MLD (reaching a nominal thickness of ∼2 nm, ∼10 nm, and ∼20 nm, respectively, determined according to the growth rate measured by ellipsometry on a spectating Si wafer) increased the monovalent selectivity of the membrane to 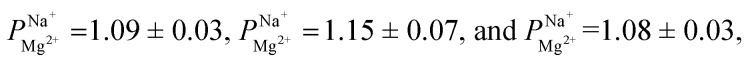 respectively. Notably, one membrane demonstrated an exceptionally high selectivity of 
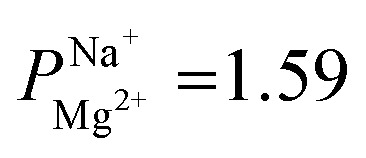
 after 25 cycles, higher than the selectivity of the PC-MVK membrane 
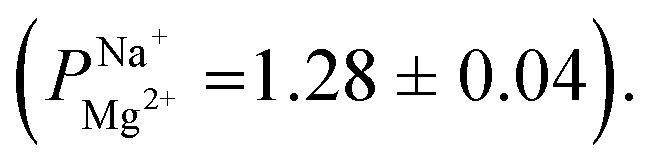
 This result reflects the potential for achieving higher selectivity by further optimizing the coating method, but are not discussed further (this result was not included in the average value calculations). Drying the uncoated PC-SK membranes in conditions similar to those used in the MLD, did not considerably affect their selectivity 
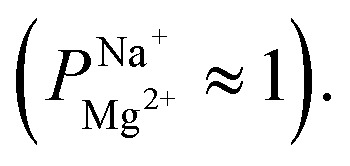
 In all ED experiments, the current efficiency for all membranes was ∼100% (Fig. S6†), indicating that the current was transferred by ion transport without side reactions (*e.g.*, water-splitting at the membrane surface). Thus, the observed selectivity between sodium and magnesium ions was solely due to the MLD coating.

**Fig. 3:**
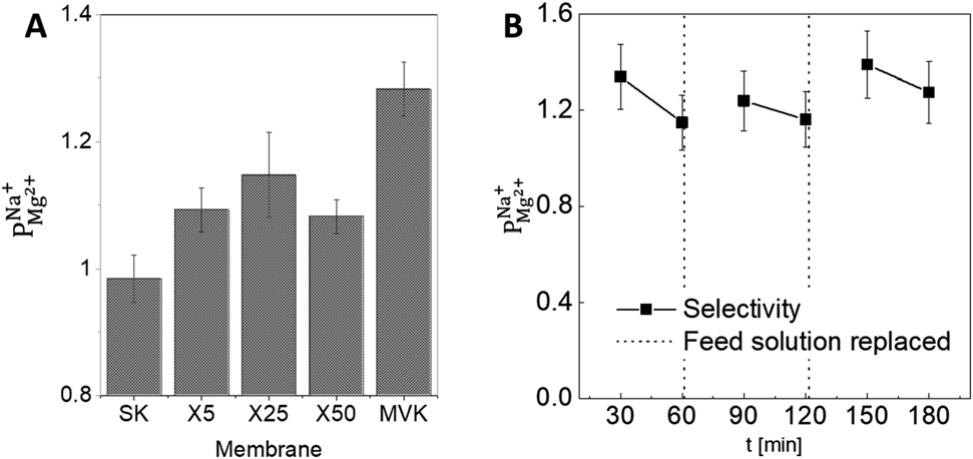
Monovalent selectivity of CEMs. (A) Selectivity after 60 min of electrodialysis, using the uncoated PC-SK membrane, PC-SK membranes coated with 5, 25, or 50 cycles of alucone MLD, and a commercial monovalent-selective PC-MVK. (B) Results for three consecutive 60 min electrodialysis experiments with the X25 alucone membrane, showing a stable performance over time. The decrease in selectivity during each single desalination experiment is due to the greater depletion of Na^+^ compared to Mg^2+^ with the selective membranes, leading to a change in their ratio in the solution and, therefore, to lower Na^+^ availability and increased Mg^2+^ transfer. Error bars are based on estimated uncertainty in concentration determination by ICP-OES.

As a preliminary test of the stability of the modified PC-SK membrane, we used the X25 membrane in three consecutive 60 min desalination experiments (Fig. 3B), using the same membrane for all experiments but refreshing the feed with newly prepared solutions. The operational performance (voltage and current) remained stable and the selectivity recovered after refreshing the feed solution. This result demonstrates the stability of the coated membrane under the tested operational conditions.

To verify that a charge-based exclusion mechanism indeed caused the observed selectivity in a coated membrane, we performed ED with the X25 membrane at pH ∼9.5, which is slightly above the IEP of the alucone layer. At this pH, the selectivity of the coated membrane was similar to that of the uncoated membrane 
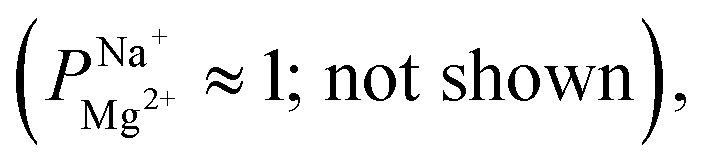
 supporting our assertion that the selectivity is mostly instilled by a charge-based exclusion mechanism, which is induced by the positively charged alucone layer and disappears when the coating loses its positive surface charge at the IEP.

The selectivity of the membrane coated by 25 MLD cycles (Fig. 3A) was higher than that of both the coatings produced by 5 and by 50 MLD cycles. Possible explanations could be suggested based on the layer morphology. As seen in Fig. 2 and mentioned before, large uncoated dimples (20–30 nm diameter) exist after 5 deposition cycles. Considering that at the ionic strength of our solution the Debye length is 1–2 nm, both Na^+^ and Mg^2+^ (having a Stokes radius of 1.84 Å and 3.47 Å, respectively^44^), could easily pass through the dimples unaffected by the coated layer. At 50 MLD cycles, the entire surface is coated, though a few cracks start to appear in the thick layer, which have a detrimental impact on selectivity. 25 MLD cycles could represent an optimum in the coating thickness: 25 MLD cycles is sufficient to coat the entire surface and instill monovalent selectivity, but ∼10 nm is not too thick that the alucone starts to be brittle. Another hypothesis could be related to the gradual narrowing of said dimples, which, as they narrow from the sides inwards, might result in the formation of channels that are sufficiently small to hinder the permeation of multivalent ions more than that of monovalent ions, thus leading to an increased selectivity around 25 MLD cycles.

In summary, PC-SK membrane coated by 25 MLD cycles produced a selectivity value of 
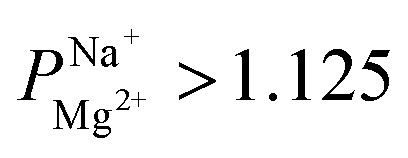
—suitable for selective brackish water desalination. However, MLD has the potential for great improvements in selectivity as gleamed from the single result of 
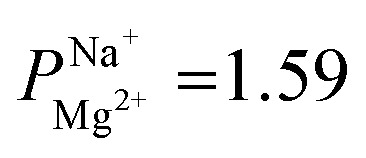
 mentioned, and further optimization of the MLD process might lead to higher selectivity values. Furthermore, MLD is a versatile coating method and various organic and inorganic components can be exchanged, which paves the path to exploring other compositions and materials with properties tailored to specific applications and potentially makes ED relevant for various other uses.

Typical non-selective CEMs have resistances of ∼2.5 Ω cm^2^.^45^ The added resistance due to the selective coating on MVS-CEMs affect the viability of their application because of the added cost of energy. Area resistance measurements from the literature (performed with 0.5 M NaCl solutions) show that deposition of a positively charged polyelectrolyte such as polyethylenimine, the most commonly used method to generate monovalent-selectivity, results in ∼2 Ω cm^2^ increase in resistance compared to the uncoated membrane.^46^ Layer by layer deposition of multiple polyelectrolyte layers (PAH/PSS) results in a similar increase in resistance,^33^ and membranes prepared by heavier crosslinking of the membrane surface tend to experience an even higher increase in resistance.^47^

**Fig. 4:**
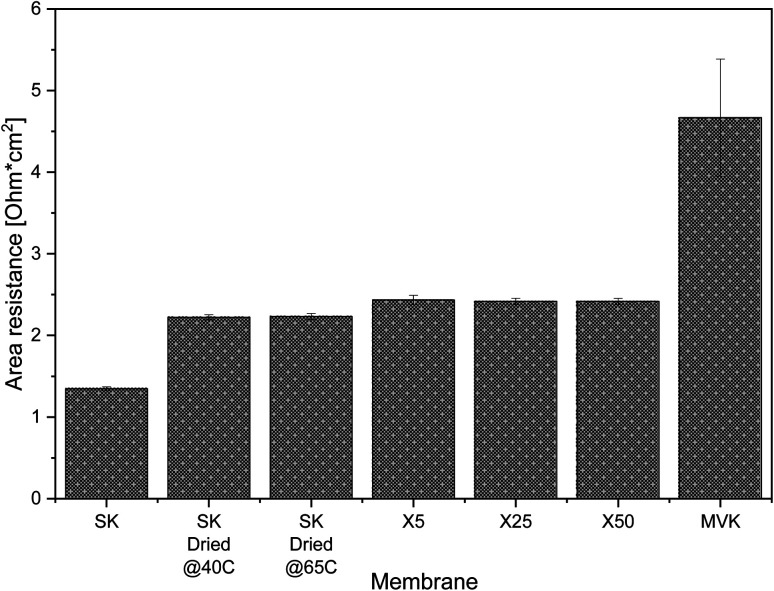
Area resistance of PC-SK membranes as-bought, after drying in the ALD reactor and after coating by 5, 25 and 50 MLD cycles, as well as resistance for commercially available monovalent-selective PC-MVK.

The resistance of our MLD-coated membranes (Fig. 4) is ∼0.2 Ω cm^2^ higher than the resistance of uncoated PC-SK that has been dried, and half the resistance of a commercially available monovalent selective PC-MVK (4.7 ± 0.7 Ω cm^2^), which was not dried. The measured resistance in the coated membranes was (within the accuracy of our measurements) identical regardless of the number of MLD cycles applied: 2.42 ± 0.04 Ω cm^2^ for membranes coated by 25 and 50 MLD cycles, and 2.43 ± 0.06 Ω cm^2^ for membranes coated with 5 MLD cycles. Resistance measurements performed in 0.1 N KCl and 0.5 N MgCl_2_ (Fig. S7†) produced higher overall resistance values but shown similar variations between membranes as in 0.5 N NaCl. This shows that the coated membranes have low added resistance and, most importantly, that the MLD coating itself adds little resistance to the base membrane. The low added resistance incurs a marginal added energy cost to a selective ED process compared to non-selective ED (∼4%), while using existing methods for instilling selectivity or a PC-MVK membrane would add at least 40% to the energy consumption in brackish water ED (see details of the calculations in Section 8 of the ESI†).

The specific resistance of the as-bought PC-SK membranes (*i.e.*, membranes that had not been dried) was 1.3 ± 0.02 Ω cm^2^. Lowering the drying temperature to 40 °C, within the manufacturers recommended operating conditions, did not lead to any changes in resistance compared to drying at 65 °C, which indicates that the drying-rewetting process itself impact the membrane resistance. This highlights the potential of using MLD to fabricate MVS-CEM's with very low resistance, *e.g.* by coating CEMs that have lower initial resistance and that were not pre-wetted, as we plan to do in future work.

## 4. Conclusions

We modified a commercially available CEM with 2–20 nm thick layers of a porous metal oxide having a high IEP using MLD—a simple, low-cost, and scalable deposition method applicable to various materials. The added layer increased the monovalent selectivity of the membrane to 
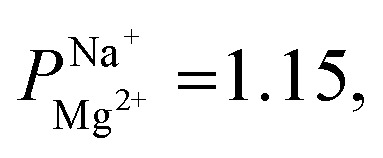
 mostly by enhancing an electrostatic repulsion of divalent ions, while causing only a minor increase (∼0.2 Ω cm^2^ in 0.5 M NaCl) in monovalent ion transport resistance. We found that the selectivity is sufficient to desalinate brackish water for potable use, allowing to maintain a sufficient amount of vital minerals in the desalinated water with a very low energy penalty due to the low resistance of the selective coating. With tens of millions of people consuming desalinated brackish water, this technology, which allows for selective desalination without a major cost increase, could be highly beneficial. We showed that the use of MLD for the deposition of ultra-thin organic–inorganic layers, performed here, for the first time on ion-exchange membranes, leads to the formation of low-resistance monovalent-selective CEM. With a negligible impact on energy cost compared to other monovalent selective coatings, MLD coatings have an impact on introducing monovalent-selective membranes to selective brackish water ED for the production of healthier and cheaper desalinated water.

Future work should address the impact of the process conditions (membrane drying) on resistance and attempt to further increase the monoselectivity of the membrane, for example by variation of the organic component of the coating, which would allow its utilization in other applications that require higher selectivity.

## Conflicts of interest

There are no conflicts to declare.

## Supplementary Material

RA-011-D0RA08725D-s001
