# Supplementary material for: Low-resistance monovalent-selective cation exchange membranes prepared using molecular layer deposition for energy-efficient ion separations
Source: RSC Adv. 2021 Jan 11;11(4):2427–36. doi: 10.1039/d0ra08725d (PMC9134019; doi:10.1039/d0ra08725d)
Supplement: RA-011-D0RA08725D-s001 [file RA-011-D0RA08725D-s001.pdf]

Electronic Supplementary Material (ESI) for RSC Advances.
This journal is © The Royal Society of Chemistry 2020

Supporting Information

Low-Resistance Monovalent-Selective Cation Exchange Membranes Prepared using Molecular Layer Deposition for Energy-Efficient Ion Separations

Eyal Merary Wormser[1,2], Oded Nir[1,*], Eran Edri[2,*]

[1]Blaustein Institutes for Desert Research, Zuckerberg Institute for Water Research, Ben-Gurion University of the Negev, Sede Boqer Campus 8499000, Israel

[2] Department of Chemical Engineering, Ben-Gurion University of the Negev, Beer-Sheva 8410501, Israel

SI contents:

1. Selectivity requirements in brackish water electrodialysis
2. Schematic of the electrodialysis system used in experiments
3. Thermal stability of the coated membrane
4. Development of the alucone MLD procedure and growth analysis
5. XPS and FTIR Spectroscopy
6. Current efficiency
7. Membrane resistances in different electrolytes
8. Estimation of impact on energy consumption

1. Selectivity requirements in brackish water electrodialysis

Brackish water varies in salinity and composition according to its source, giving advantage to tunable membranes that could have selectivity fitting specific requirements. As an example, the brackish groundwater of the Israel's Negev region contains approximately 1000 mg/L of Na^+ and 100 mg/L of Mg^{2+}. In addition, drinking water regulations and standards vary, with no clear worldwide standard for permitted or required Na^+ [1] or Mg^{2+} [2] set by the world health organization (WHO). Most freshwater contains Na^+ in concentrations of <20 mg/L, and water becomes noticeably salty in taste with Na^+ concentrations above ~200 mg/L. Water is a minor source of Na^+ in terms of nutrition; diets tend to include too much sodium in western diets, and sometimes lack sodium in non-western ones. in the US, tap water was found to contain ~1-400 mg/L Na^+.[1] Overall, no single standard value could be chosen. A reasonable target value of <100 ppm sodium has been chosen for demonstration. In Israel, the Ministry of Health has considered adding magnesium to desalinated drinking water in concentrations of 20 mg/L.[3] We performed an estimate on required selectivity based on target values of <100 mg/L sodium and >20 mg/L Mg^{2+}:

$$P_{Mg^{2+}}^{Na^+} = \frac{(1000\ mg/L - 100\ mg/L)/1000\ mg/L}{(100\ mg/L - 20\ mg/L)/100\ mg/L} = 1.125$$

Thus, $P_{Mg^{2+}}^{Na^+} > 1.125$ could be considered sufficient for brackish water to drinking water desalination, in this case. This value could be lower or higher given different initial and target concentrations.

2. Schematic of the electrodialysis system used in experiments

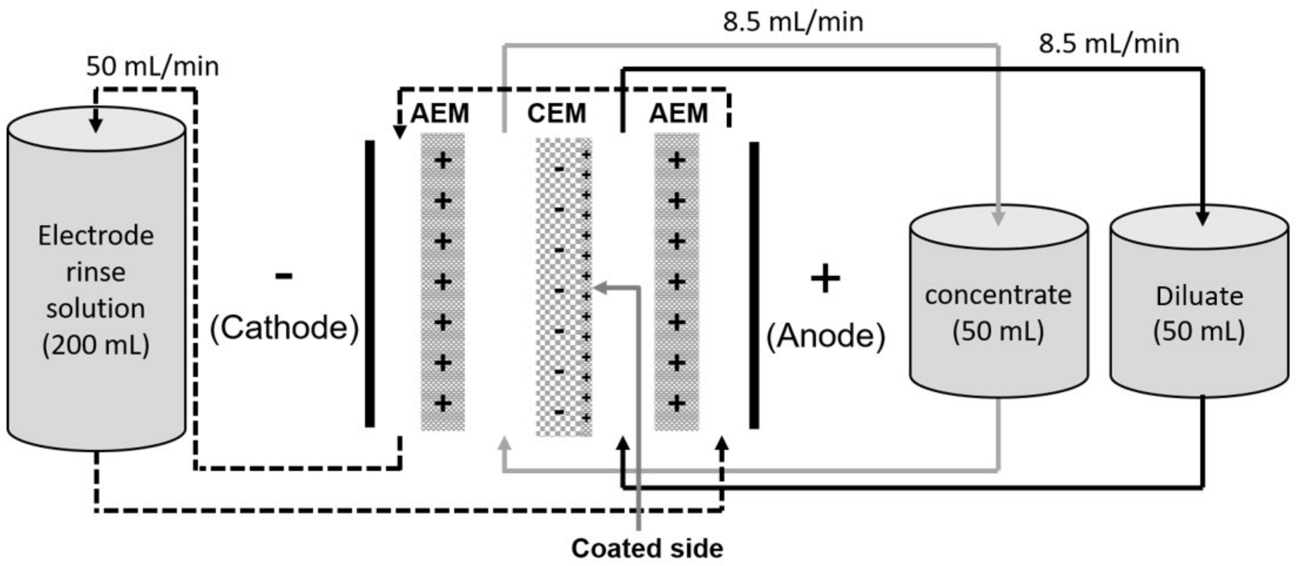

Figure S1: Schematic illustration of the electrodialysis system, volumes, and flow rates used in the desalination experiments. An applied electric potential on the electrodes leads to the migration of ions. In the diluate compartment, cations permeate through the CEM and anions through the AEM. In the concentrate, channel cations encounter the negatively charged AEM and fail to permeate, and anions are similarly blocked by the CEM, leading to a concentration of ions in the concentrate channel. In experiments with MVS-CEMs, the monovalent-selective layer faces the diluate stream. The layer has lower permeability to multivalent ions, reducing their removal rate from the diluate channel.

3. Thermal stability of the coated membrane

DSC analysis was performed to check the thermal stability range of the PC-SK membrane, using a 2.28 mg sample air dried for several days. The results (Figure S1) showed glass transition at 85 °C, with possible onset at 70–80 °C. Based on these results, 65 °C was decided to be the highest safe temperature for performing ALD/MLD without altering the membrane structure. Melting/decomposition onset was at ~190 °C. The change observed in the 30–50 °C range is an artifact due to the initial stabilization of the system.

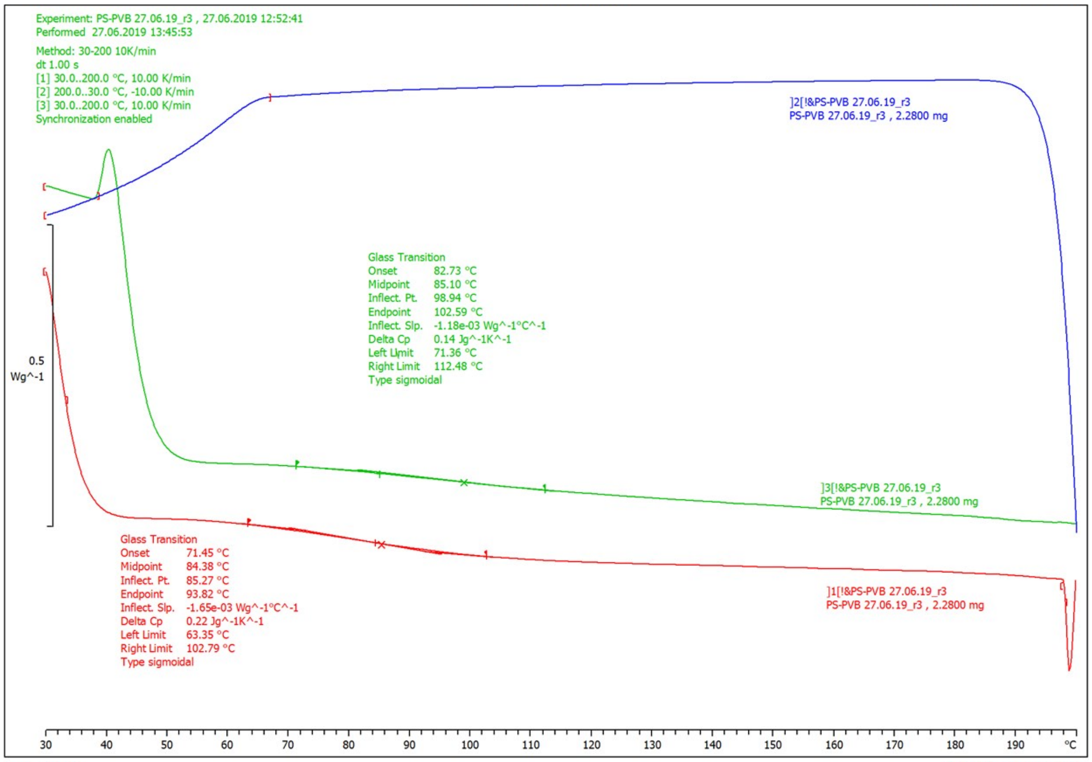

Figure S2: DSC results, showing weight loss vs. temperature, scanned at 10 °C/min. The bottom (red) line shows first a heating cycle from 30–200 °C, top (blue) cooling back to 30 °C, and middle (green) the 2nd heating cycle, showing glass transition around ~85 °C.

4. Development of alucone MLD procedure and growth analysis

Several techniques were used to develop the MLD procedure. ***In-situ QCM*** (an example using the procedure used in our experiments is shown in Figure S3A) showed the stepwise growth and gave a measure for the overall growth rate. It also helped to establish the stabilization time after each reagent dose, which was monitored through the change in pressure in the reactor. This allowed the selection of appropriate reagent exposure times (eventually decided to be 21 ms for the TMA and 1 s for the EG) and purge times between subsequent reagent exposures, set to be 60 s. Following deposition, ellipsometry (Figure S3B) was used to measure the thickness of alucone deposited on Si substrates, and by performing the deposition with a membrane present in the reactor we could see the effect of reagents adsorbed and released from the membrane, leading to an increased growth rate on the Si substrate – 3.8 Å/cycle for the alucone with the membrane vs 2.2 Å/cycle without it. Refractive index for the alucone layer was assumed at a constant 1.5, based on ref. [4] A rough, purely geometric analysis (Figure S3D) shows that a fully extended chain grown by an alucone deposition cycle could have a thickness of up to ~8.4 Å. The fact that the growth rates measured by ellipsometry were lower than that indicates that a monolayer-by-monolayer growth is plausible. As ellipsometry was not feasible on the membranes, the amount of Al on the membranes was quantified by EDS. EDS was performed at the same conditions in all samples (accelerating voltage, current, and working distance), and is assumed to have a similar penetration depth and sampling volume of the electron beam. This depth is estimated to be >1 µm, much higher than the thickness of the deposited layer, meaning that a similar volume of the substrate membrane was sampled each time. We assume the sulfur concentration is homogenous and fixed in the membrane, therefore the Al/S ratio qualitatively indicates the Al concentration in the thin layer at the membrane surface. The results (Figure S3C) show that the Al/S ratio grows linearly and that the amount of Al added in each cycle is constant in the ALD and MLD procedures.

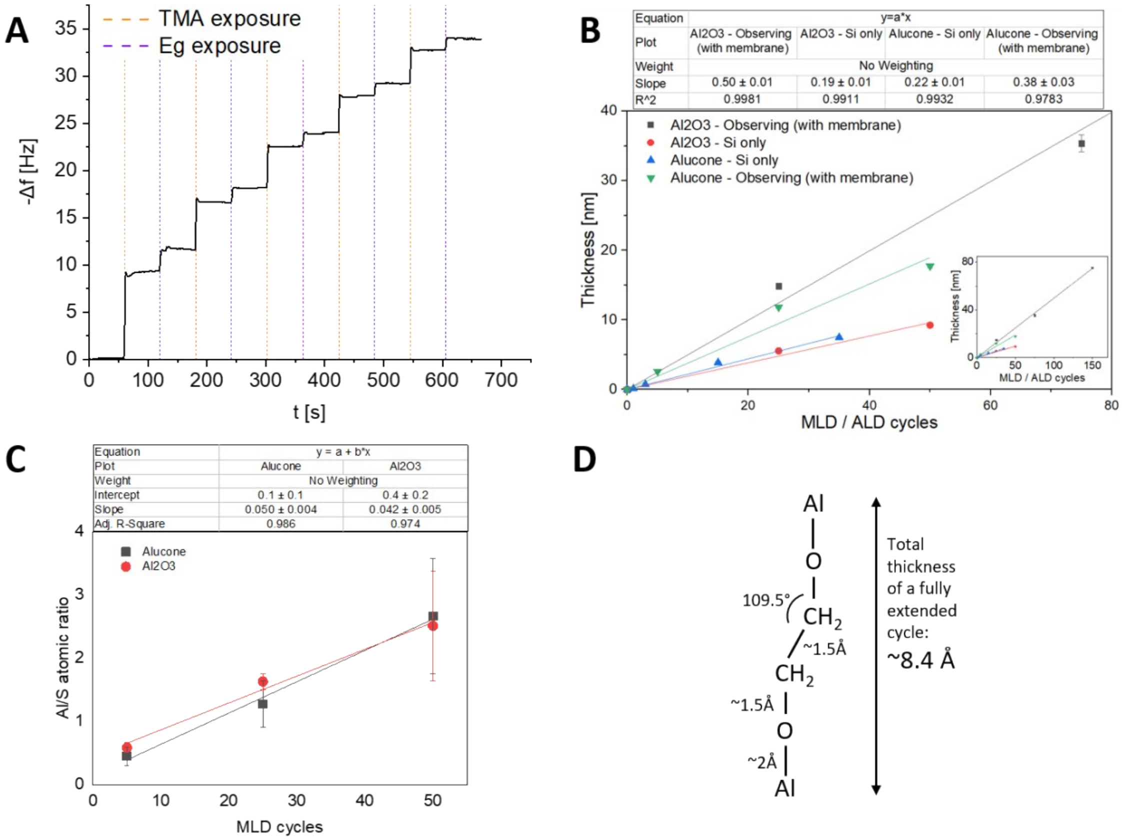

Figure S3: Alucone growth. (A) Change in QCM frequency during five MLD cycles. (B) Ellipsometry results showing thickness and linear regression fits for average growth rate of alucone MLD and Al_2O_3 ALD, with and without membranes present in the reactor. Inset is an expansion up to 150 cycles. Error bars indicate standard deviation. (C) Al/S atomic ratio measured by EDS (measured on an area of several square millimeters). Sulfur stems from sulfonate groups in the membrane. Sampling depth is expected to be >1 µm, meaning that the amount of sulfur is insensitive to the thin (<30 nm) Al_2O_3 and alucone coatings, and the ratio reflects the amount of Al in the alucone. Al concentration grows in a linear trend with MLD cycles and in equal rate in ALD and MLD. (D) Rough geometric sketch of ethylene glycol molecule illustrating the maximum increase in thickness that could stem from alucone single monolayer deposition.

5. Spectroscopy

We performed XPS and ATR-FT-IR to better understand the chemical structure of the deposited layer. Primary conclusions are included in the main text; the full spectra and further discussion are presented here.

FTIR. Through ATR-FT-IR analysis, we find several FT-IR bands in the modified membrane that are not apparent in the pristine membrane (Figure S6). These bands are centered around ~1598 cm^{-1} and ~850 cm^{-1}. The former is attributed to vinyl ether groups, while the latter is a convolution of several bands, including vinyl ether asymmetric vibration and Al-O vibration. Additionally, we found a small shift in the ~1250 cm^{-1} band and no indication of C=O groups by FT-IR (at 1700-1750 cm^{-1}). It is well documented that the alucone layers go through chemical and structural transformation after exposure to ambient conditions, water, or heat.[4,5] Dameron et al. used XPS and FT-IR to study the composition of alucone deposited on hard substrates. They noted the formation of C=O groups in the alucone layer after exposing the layers to ambient for 24 h. Recently, Van de Kerckhove et al., studying the transformation behavior of alucones, showed that, after several days of exposure to ambient, the alucone layer contains vinyl ether functional groups, which are mostly removed after etching in water. Therefore, our XPS and FT-IR observations are in agreement with previous observations of the transformation of alucone in ambient or water.[4,5] Specifically, our results indicate the formation of vinyl ether functional groups, such as CH2=CH-O-Al in the alucone layer, which is accompanied by a dehydration reaction. Furthermore, we did not find direct evidence for a reaction of the MLD precursors with the membrane.

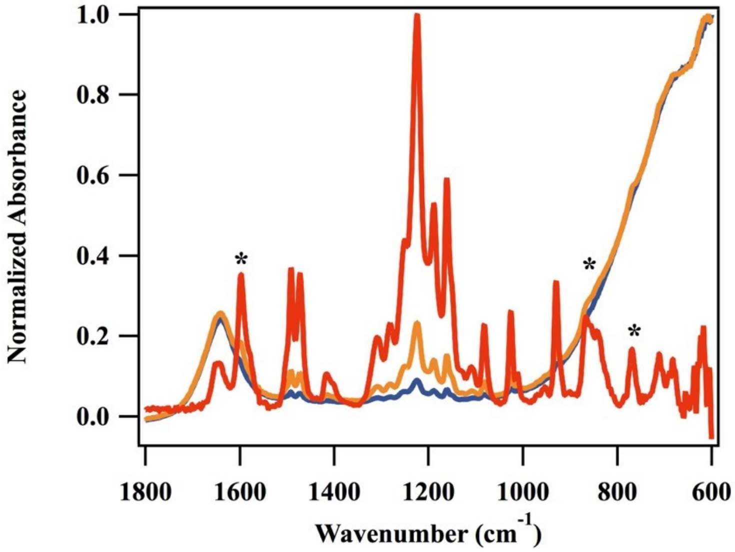

Figure S4. ATR-FT-IR spectra of pristine (blue) and modified (orange) PC-SK membrane. Weighted difference spectrum is shown in red (modified minus pristine; weighing factor = 0.9462). Most of the bands in 900-1580 cm^{-1} appear in both pristine and modified membranes spectra, and the membrane-related peak enhancement is attributed to differences in the dielectric constant at the interface of the membrane-ATR crystal. However, peaks designated with an asterisk appear only in the modified membrane and are attributed to the alucone layer (see main text). Spectra were measured with a Ge ATR crystal at 60° incident and reflection beam angles at a resolution of 4 cm^{-1} with a DTGS detector. Background spectrum was air. Both instrument and ATR were continuously purged with dry N_2.

XPS spectra of alucone-coated membranes before and after exposure to water were measured (Figure S5). Atomic composition of the coating was 21% Al, 34% C, and 45% before wetting and 16% Al, 45% C, and 39% O after water exposure. The amount of carbon was likely influenced by the presence of adventitious carbon, distorting the absolute composition but not the O/Al atomic ratio. O/Al atomic ratio was 2.1 after deposition, rising to 2.4 after wetting. Ideal alucone ($Al_2(OCH_2CH_2O)_3$) should theoretically have an O/Al ratio of 3 and ideal Al_2O_3 – a ratio of 1.5, meaning that the deposited layer does not follow the ideal alucone structure. The lower-than-expected O/Al ratio could be explained by the existence of unreacted methyl groups and Al_2O_3-like regions, arising from hydrolysis and rearrangement of the structure in ambient air or water. The rise in O/Al ratio does not support conversion to Al_2O_3 due to water exposure, as sometimes reported,[6,7] but fits the existence of unreacted methyl groups that reacted with water. The C1s peak of an as-prepared PC-SK membrane coated with 50 cycles of alucone (a thickness that is sufficient to suppress the S2p peak from sulfonate groups of the membrane, as seen in the inset of Figure S5A) shows binding energies corresponding to C-C and C-H bonds (~285 eV), and C-O bonds (a shoulder at ~286.1 eV). After soaking the membrane in water for 2 h, we observed an increase in intensity of the 285 eV peak alongside the emergence of a new peak at ~288.7 eV. We also find small shifts of Al2p and O1s peaks to higher binding energies (0.4 and 0.2 eV, respectively). We attribute the latter changes to a decrease in hydroxide and oxyhydroxide species content (e.g., $AlO(OH)_2$ or $Al(OH)_3$).

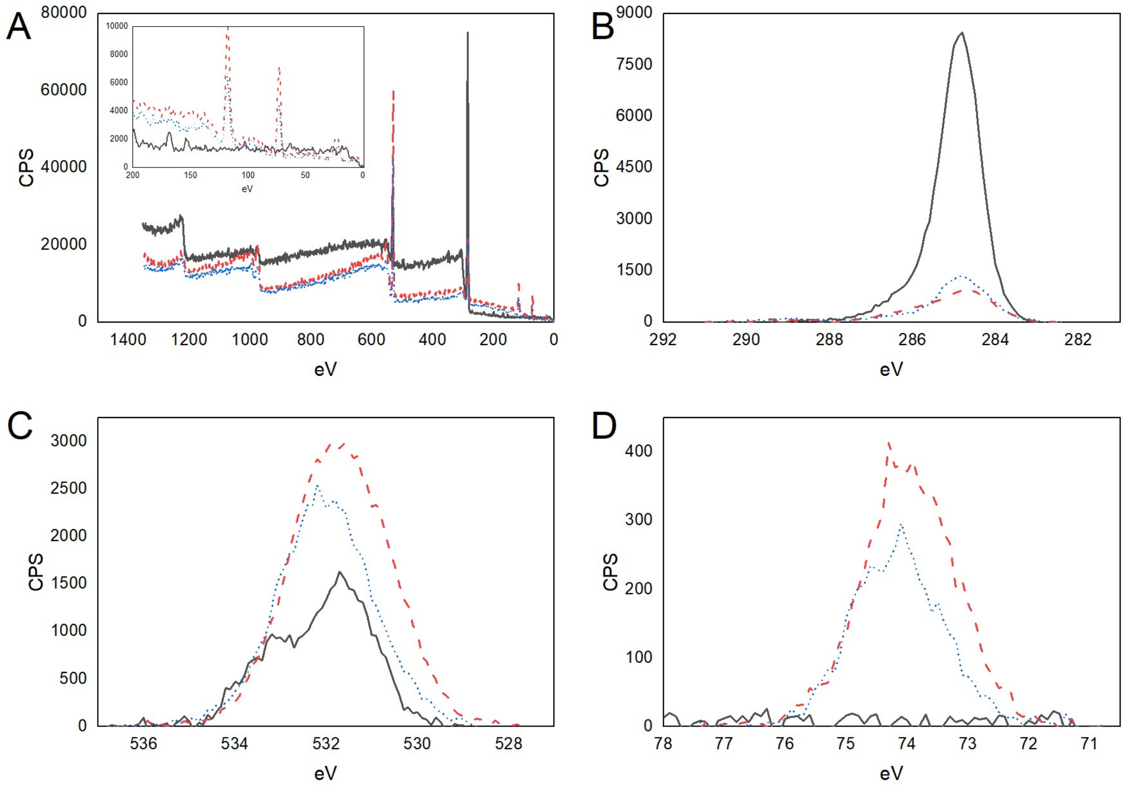

Figure S5: (A) XPS spectra of uncoated membrane (black line), an alucone coated membrane before (red dashed line) and after wetting for 24 h (blue dotted line; 50 MLD cycles). Inset shows magnification of the same spectra in the 0-200 eV region. Photoelectric line of C1s (B), O1s (C) and Al2p (D) peaks with background subtracted. No S2p peak was observed after coating, signifying that the layer was thick enough to suppress the S2p peak at 168 eV from the coated membrane (which contains sulfonate groups).

6. Current efficiency

Faradaic efficiency (FE) was calculated for all ED experiments using the equation:

$$FE = \frac{\sum X_i n_i z_i}{t * i/F}$$

The numerator represents the total number of mol-equivalents transferred: percentage transport for each cation (X_i) times the number of moles of the species in the feed solution (n_i) and the valence of that species. The denominator shows the total number of electrons transferred through the system – with t being the desalination time, I the current, and F Faraday's constant (= 96500 C/mol). Results are shown in Figure S6.

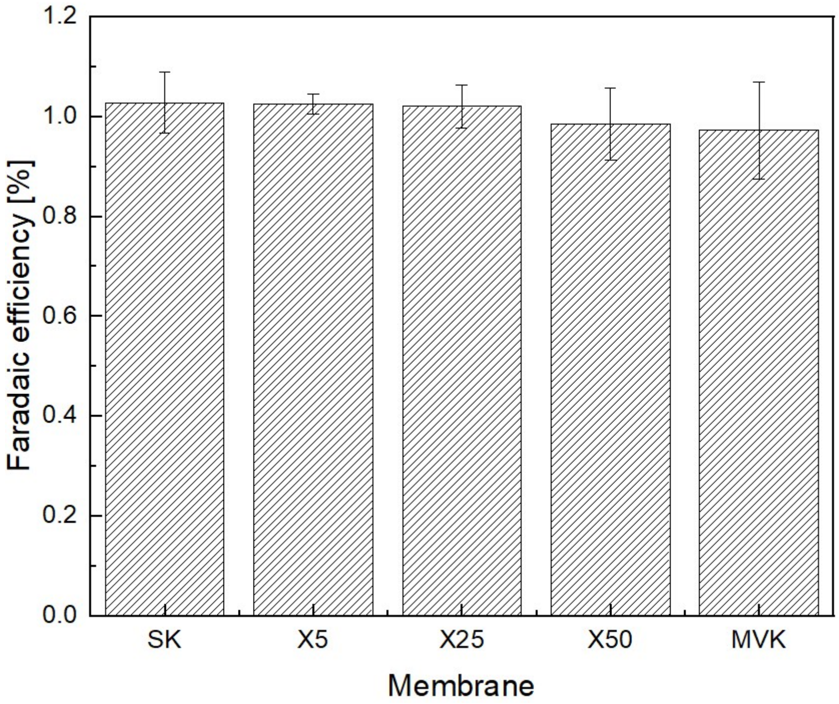

Figure S6: Faradaic efficiency in electrodialysis experiments with all membranes was ~1, indicating that all current was transferred through ion transfer and not by water splitting at the membrane surface. Values and errors represent averages and standard deviations for at least three experiments with each membrane type.

7. Membrane resistances in different electrolytes

Membrane resistance was measured in 0.5 N MgCl2 and 0.1 N KCl in addition to the 0.5 N measurements included in the main text. As the measurements in 0.5 N NaCl are the most relevant for comparison to the literature, they were included in the main text. Measurements in 0.1 N KCl show the same trends as in 0.5 N NaCl with exaggerated resistances. Interestingly, even though the MLD-coated membranes show higher selectivity in ED experiments than the dried, uncoated PC-SK membranes, this does not express itself in the resistance measurements and resistance in 0.5 N $MgCl_2$ is roughly identical before and after coating. Additionaly, despite the selectivity of the MVK being higher in ED than that of the coated membranes, the ratio between resistance in $MgCl_2$ and in NaCl is higher in the coated membranes (~7) than in the PC-MVK (~4.6), which features a similar ratio as the uncoated, un-dried PC-SK (~4.4).

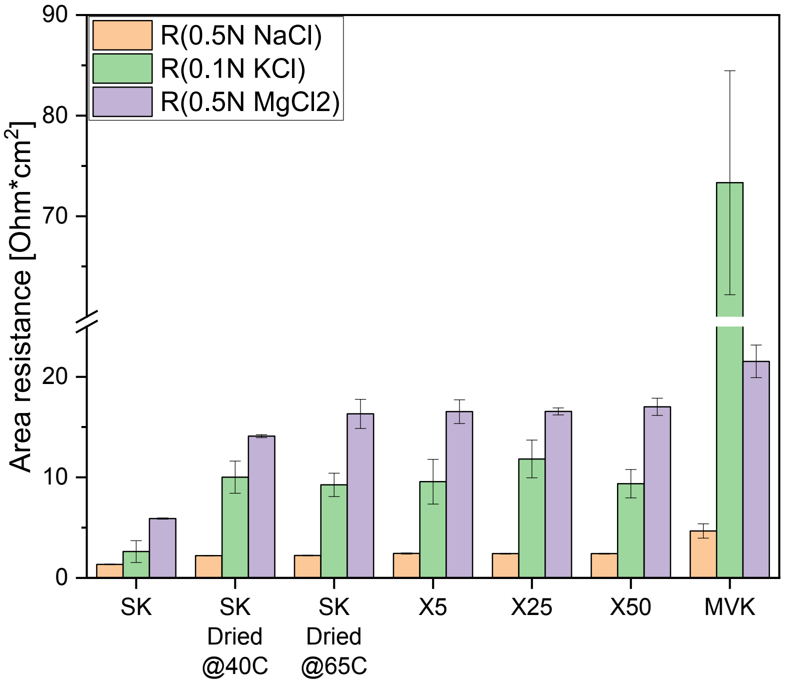

Figure S7: Area resistance of membranes in different electrolyte solutions.

8. Estimation of impact on energy consumption

To quantify the energy saved by using a lower-resistance selective membrane, we use the model described by H. Strathmann's "Assessment of Electrodialysis Water Desalination Process Costs".[9] Energy consumption in commercial scale-ED installations is dominated by the electric energy invested in transferring ions across the ion exchange membranes from the diluate to the concentrate. Energy invested in pumping is mostly relevant at lower salinities and other energy requirements, such as those for electrode reactions, are generally neglected.[8] The specific energy cost for desalinating a unit volume product is proportional to the cell-pair area resistance, which can be expressed as:

$$R = \frac{\Delta \cdot \ln\left(\frac{C_s^{fd} C_s^{c}}{C_s^{fc} C_s^{d}}\right)}{\Lambda_s\left(C_s^{fd} - C_s^{d}\right)} + r^{am} + r^{cm} \#(eq\ 2)$$

R being the cell pair area resistance; Δ the cell thickness; C_s^{fd} and C_s^{fc} the equivalent concentrations at the feed for the diluate and concentrate, respectively; C_s^{d} and C_s^{c} the outlet concentrations of the diluate and concentrate, respectively; Λ_s the equivalent conductivity of the solution (which can be approximated as that of a solution within the operation salinity range without inducing great error)[9]; and r^{am} and r^{cm} the area resistances of the AEMs and CEMs, respectively. Equation 34 in Strathmann's paper with a recovery ratio of 0.5 (equal diluate and concentrate volumes, as used in this work) reduces to:

$$C_s^{c} = 2C_s^{fd} - C_s^{d} \#(eq\ 3)$$

And

$$C_s^{fc} = C_s^{fd} \#(eq\ 4)$$

C_s^{d} can be also be expressed in terms of desalination percentage, X:

$$C_s^{d} = (1 - X) \cdot C_s^{fd} \#(eq\ 5)$$

Λ_s for NaCl was estimated at 25°C using the Debye-Hückel-Onsager equation :[10]

$$\Lambda_s = 126.39 - 89.14 \cdot \sqrt{C} \#(eq\ 6)$$

The ratio of energy consumption between processes using different CEMs can be expressed as:

$$\frac{E_1}{E_2} = \frac{r^{sol} + r^{am} + r_1^{cm}}{r^{sol} + r^{am} + r_2^{cm}} \#(eq\ 7)$$

Ratios are not bound to specific process-dependent factors, such as membrane areas, flow rates, number of cell pairs, etc.

r^{sol}, the resistance posed by the solution, approximately equal to:

$$r^{sol} = \frac{\Delta \cdot \ln\left(\frac{1+X}{1-X}\right)}{\left(126.4 - 89.1\sqrt{C_s^{fd}}\right)\left(X \cdot C_s^{fd}\right)} \#(eq\ 8)$$

with r_1^{cm} and r_2^{cm} being the area resistances of the two CEMs compared. A desalination percentage of 90% was chosen. r^{am} was taken as 2 Ω·cm^2, a typical value for commercial IEMs.[8] Cell pair thickness was taken, as used in this work, ~1 mm. Comparisons were performed for brackish water at similar salinity to that used in this work ($C_s^{fd} = 0.05M$), seawater ($C_s^{fd} = 0.65M$), and brine ($C_s^{fd} = 1.3M$) salinities.

With these parameters, a comparison of the X25 membrane from this work ($r^{cm} = 2.4\ \Omega \cdot cm^2$) to the MVK membrane ($r^{cm} = 4.7\ \Omega \cdot cm^2$) predicts that using the MVK membrane in large-scale ED would lead to approximately 46% higher energy consumption in brackish water ED than using the X25 alucone membranes from this work, or 51% higher for the case of seawater and brine ED. A similar comparison of a non-selective CEM ($r^{cm} = 2.5\ \Omega \cdot cm^2$) compared to selective ones with MLD coating with $0.2\ \Omega \cdot cm^2$ added resistance ($r^{cm} = 2.7\ \Omega \cdot cm^2$) and with state-of-the-art monovalent selective coatings with $2\ \Omega \cdot cm^2$ added resistance (a total resistance of 4.5 Ω·cm^2)[11,12,13] predicts alucone-coated membrane will increase the total energy consumption by ~4% compared to non-selective CEMs, whereas state-of-the-art monovalent selective coatings would increase energy consumption by ~40% (for brackish water ED) and up to 44% in higher salinities.

References

1 World Health Organisation, *Sodium in Drinking-water Background document for development of WHO Guidelines for Drinking-water Quality*, 2003.

2 World Health Organisation, *Calcium and Magnesium in Drinking-water - Public health significance*, 2009.

3 Knesset research and information center, *Addition of magnesium to desalinated water*, 2012.

4 A. A. Dameron, D. Seghete, B. B. Burton, S. D. Davidson, A. S. Cavanagh, J. A. Bertrand and S. M. George, *Chem. Mater.*, 2008, **20**, 3315–3326.

5 K. Van De Kerckhove, M. K. S. Barr, L. Santinacci, P. M. Vereecken, J. Dendooven and C. Detavernier, *Dalt. Trans.*, 2018, **47**, 5860–5870.

6 L. Ghazaryan, E.-B. Kley, A. Tünnermann and A. Viorica Szeghalmi, *J. Vac. Sci. Technol. A Vacuum, Surfaces, Film.*, 2013, **31**, 01A149.

7 X. Liang, B. W. Evanko, A. Izar, D. M. King, Y. Jiang and A. W. Weimer, *Microporous Mesoporous Mater.*, 2013, **168**, 178–182.

8 H. J. Lee, F. Sarfert, H. Strathmann and S. H. Moon, *Desalination*, 2002, **142**, 267–286.

9 H. Strathmann, in *Proceedings of the International Conference on Desalination Costing, Lemassol, Cyprus, December 6-8, 2004*, 2004, pp. 32–54.

10 P. Vanýsek, *Handb. Chem. Phys.*, 2012, 5–76.

11 T. Sata and R. Izuo, *J. Memb. Sci.*, 1989, **45**, 209–224.

12 T. Rijnaarts, D. M. Reurink, F. Radmanesh, W. M. de Vos and K. Nijmeijer, *J. Memb. Sci.*, 2019, **570**–**571**, 513–521.

13 G. S. Gohil, V. V. Binsu and V. K. Shahi, *J. Memb. Sci.*, 2006, **280**, 210–218.
